# Deep cervical stromal invasion predicts poor prognosis in patients with stage II endometrioid endometrial cancer: a two-centered retrospective study

**DOI:** 10.3389/fonc.2025.1450054

**Published:** 2025-02-27

**Authors:** Wenyu Shao, Yu Xue, Zhiying Xu, Jun Guan, Huaying Wang, Xiaojun Chen, Yulan Ren

**Affiliations:** ^1^ Department of Gynecology, Obstetrics and Gynecology Hospital of Fudan University, Shanghai, China; ^2^ Department of Gynecologic Oncology, Fudan University Shanghai Cancer Center, Shanghai, China; ^3^ Department of Oncology, Shanghai Medical College, Fudan University, Shanghai, China; ^4^ Department of Gynecology, Tongji University Tenth People’s Hospital, Shanghai, China

**Keywords:** endometrial neoplasms, depth of cervical stromal invasion, radiotherapy, adjuvant, recurrence free survival, disease-specific survival

## Abstract

**Objective:**

To evaluate the impact of depth of cervical stromal invasion (CSI) on the prognosis of International Federation of Gynecology and Obstetrics (FIGO) stage II endometrioid endometrial cancer (EEC).

**Methods:**

Patients with FIGO stage II EEC confirmed by postoperative histopathology and consecutively admitted to the Obstetrics and Gynecology Hospital of Fudan University and Fudan University Shanghai Cancer Center between 2008 and 2017 were included in this study and reviewed retrospectively.

**Results:**

Two hundred and ninety-seven patients were included in this study. There were 253 (253/297, 85.2%)patients with superficial (<50%) and 44 (44/297, 14.8%) cases with deep (≥50%) CSI. The median follow-up time was 75.0 months (range: 5-175 months). Patients in the ≥50% CSI group had a poorer prognosis compared to the <50% CSI group (recurrence-free survival [RFS]: adjusted hazard ratio [aHR] = 6.077, 95% Confidence Interval [CI]: 2.275-16.236, disease-specific survival [DSS]: aHR = 7.259, 95% CI: 2.546-20.695). Deep CSI was an independent predictor of local recurrence (aHR=5.537, 95% CI: 1.804-16.991). Post operative external beam radiation therapy (EBRT) was correlated with a reduced risk of recurrence (aHR = 0.288, 95% CI: 0.097-0.859).

**Conclusion:**

Deep CSI is a poor prognostic factor for RFS and DSS in patients with FIGO stage II EEC. Postoperative EBRT can improve both RFS and DSS. Those findings imply that a detailed pathological report on the depth of CSI would be helpful in better understanding its impact on prognosis and selecting an appropriate postoperative treatment for the patient.

## 1 Introduction

Endometrioid endometrial cancer (EEC) is the most common type of endometrial cancer (EC), and its incidence continues to increase with rising population aging and obesity (1). Cervical stromal involvement is a common manifestation of EEC invasion and is associated with an increased risk of lymph node metastasis (2–5) and disease recurrence. Studies have shown that patients with EEC and cervical stromal invasion (International Federation of Gynecology and Obstetrics [FIGO] 2009 stage II) who underwent simple hysterectomy (SH) combined with radiotherapy achieved a similar prognosis to those who underwent radical hysterectomy (RH) (6–9).

However, the depth of cervical stromal invasion (CSI) varies in FIGO stage II EEC. It is not known whether FIGO stage II EEC with superficial or deep CSI has a varying or similar impact on patient prognosis. Only a few studies have addressed this question and have yielded conflicting results (10–14). Ferriss et al. studied 85 cases of FIGO stage II EEC and found deep (inner two-thirds vs. superficial/outer one-third) CSI to be an independent predictor of overall patient survival (11), while other studies have reported no difference in survival or recurrent outcomes between superficial and deep CSI in patients with FIGO stage II EEC (13, 15, 16).

Here, we conducted a two-centered retrospective study to evaluate the impact of depth of cervical stromal invasion on the prognosis and recurrent characteristics of patients with FIGO stage II EEC.

## 2 Materials and methods

### 2.1 Study population

A total of 3,249 EC patients underwent surgery at the Obstetrics and Gynecology Hospital of Fudan University (n=1,653) and Fudan University Shanghai Cancer Center (n=1,596) between January 2008 and December 2017 were screened for inclusion in this study. An institutional review board (IRB) approval was obtained from both hospitals (IRB Approval Numbers: 2023-29 and 091078-4).

The inclusion criteria were as follows: 1) no evidence of extrauterine metastasis by imaging examination before the operation (chest computed tomography [CT], pelvic and abdominal enhanced CT or magnetic resonance imaging); 2) received comprehensive surgical staging (total hysterectomy or radical hysterectomy (RH) or modified RH, and bilateral salpingo-oophorectomy, pelvic lymphadenectomy or sentinel lymph node biopsy with or without para-aortic lymphadenectomy or sampling; 3) FIGO stage II EEC diagnosed by postoperative pathological examination; 4) received standard postoperative adjuvant therapy; 5) follow-up data is available.

### 2.2 Surgery and postoperative pathology

RH was defined as the excision of the uterus with the parametrium and 3 cm of the upper vagina. Modified radical hysterectomy (mRH) was defined as the excision of the uterus with the cardinal ligament and 1.5-2 cm of the upper vagina (17). The pathological staging followed the FIGO 2009 guidelines (18). The depth of cervical stromal invasion (CSI) (deep [≥50%] or superficial [< 50%] was reviewed by senior pathologists.

### 2.3 Data collection

The clinical and pathological characteristics of the patients were collected, including age at diagnosis, menopausal status, and body mass index (BMI). Obesity was defined as BMI ≥30 kg/m (19). Data regarding type of surgery, postoperative pathological report (20–22), adjuvant treatment and follow-up were also collected. Information pertaining to post-operative adjuvant therapy was obtained from the medical records. Status of recurrence or survival was acquired from patient follow up medical records, institutional cancer registries or by telephone follow-up. The patients were followed up until December 31^st^, 2022.

### 2.4 Statistical methods

Recurrence-free survival (RFS) and locoregional recurrence-free survival (LRFS) values were calculated from the date of surgery to the date of diagnosis of recurrence or last contact for the recurrence-free patients. Locoregional recurrence was defined as vaginal and/or pelvic recurrence. Disease-specific survival (DSS) was calculated from the date of surgery to the date of death caused of EC or last contact.

Student’s t-test was used for the comparison of normally distributed continuous data, while the Mann-Whitney U test was used for non-normally distribution data. Regarding categorical variables, the chi-square test (Pearson chi-square and Pearson exact chi-square tests) was used to compare the proportions between the groups. The Kaplan-Meier method was used to estimate cumulative survival rates, with comparisons computed by the log-rank test. The Cox proportional hazards model was used for multivariate analysis of survival. Hazard ratios (HR) and 95% confidence intervals (CI) were calculated. A P value <0.05 was identified as statistically significant. Statistical analysis was performed using Statistical Package for the Social Sciences (SPSS) software, version 26.0.

## 3 Results

### 3.1 Demographic and clinicopathological characteristics

**Figure 1:**
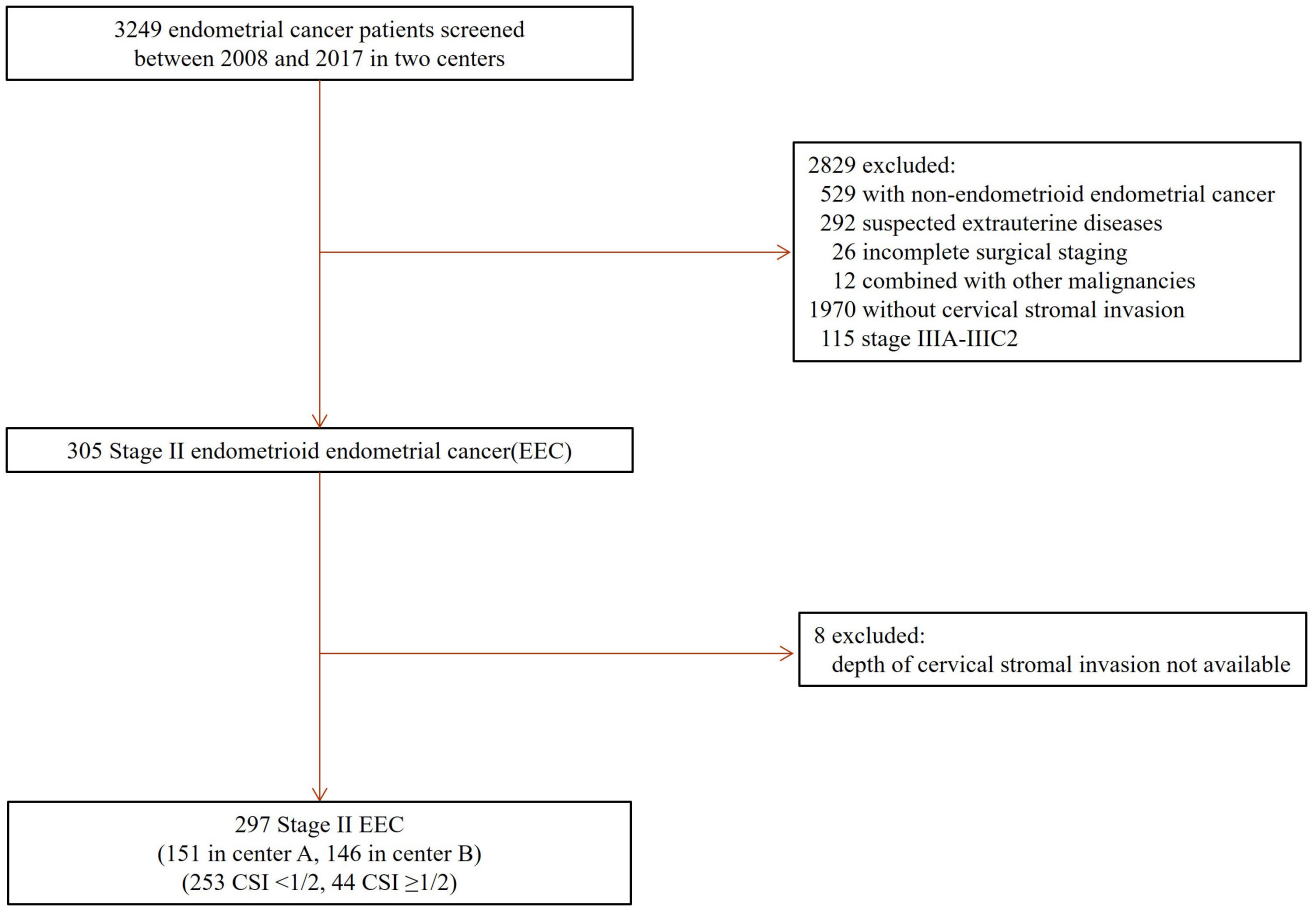
Patient selection. EEC, endometrioid endometrial cancer, CSI, cervical stromal invasion, Stage II, International Federation of Gynecology and Obstetrics (FIGO) 2009 staging classification.

Two hundred and ninety-seven patients who met all the inclusion criteria were included in the study (**Figure 1**). There were 253 (253/297, 85.2%) patients having superficial (<50%) and 44 (44/297, 14.8%) with deep (≥50%) CSI. The overall median follow-up time was 75.0 months (range: 5-175 months), while it was 72.0 months (range: 5-175months) for the superficial and 94.0 months (range: 7-148 months) for the deep CSI group.

The median age was 53 years (range: 28-77 years). Forty-seven patients (47/297, 15.8%) had a BMI ≥ 30 kg/m^2^. Sixty-two (62/297, 20.9%) patients were treated with SH and 235 (235/297, 79.1%) with RH or mRH. There were 60 (60/297, 20.2%) patients exhibiting lymph vascular space invasion (LVSI). The majority of cases 197 (197/29, 66.3%) received adjuvant treatment, with 144 (144/297, 48.5%) cases receiving external-beam radiation therapy (EBRT) with or without concurrent chemotherapy. No patient received vaginal brachytherapy (VBT) (**Table 1**).

**Table 1:** Demographic and clinicopathological characteristics of patients.

Variable	N=297	CSI <1/2	CSI ≥1/2	*P*
		(N=253)	(N=44)	
Median age (years) (range)	53.0 (28-77)	53.0 (28-77)	52.5 (32-77)	
Age (years) (%)				
<60	236 (79.5)	199 (78.7)	37 (84.1)	0.410
≥60	61 (20.5)	54 (21.3)	7 (15.9)	
BMI (kg/m^2^) (%)				
<30	250 (84.2)	212 (83.8)	38 (86.4)	0.666
≥30	47 (15.8)	41 (16.2)	6 (13.6)	
Type of surgery (%)				
SH	62 (20.9)	57 (22.5)	5 (11.4)	0.093
mRH/RH	235 (79.1)	196 (77.5)	39 (88.6)	
Surgical approach (%)				
Laparotomy	168 (56.6)	143 (56.5)	25 (56.8)	0.971
Laparoscopy	129 (43.4)	110 (43.5)	19 (43.2)	
Grade (%)				
G1-G2	261 (87.9)	224 (88.5)	37 (84.1)	0.404
G3	36 (12.1)	29 (11.5)	7 (15.9)	
Depth of MI (%)				
<1/2	193 (65.0)	**181 (71.5)**	12 (27.3) *	**0.000**
≥1/2	104 (35.0)	**72 (28.5)**	32 (72.7) *	
LVSI (%)				
No	237 (79.8)	**212 (83.8)**	25 (56.8) *	**0.000**
Yes	60 (20.2)	**41 (16.2)**	19 (43.2) *	
Adjuvant treatment (%)				
Observe	100 (33.7)	**96 (37.9)**	4 (9.1) *	**0.000**
Chemotherapy	53 (17.8)	48 (19.0)	5 (11.4)	
EBRT ± con-chemo	144 (48.5)	**109 (43.1)**	35 (79.5) *
Median follow-up time (month)	75.0 (5-175)	**72.0(5-175)**	**94.0(7-148)**	**0.016**

Values are presented as mean ± standard deviation (range), number (%), or median (range).

Bold values, there is statistically significant differences between groups. CSI, cervical stromal invasion; BMI, body mass index; Depth of MI, depth of myometrial invasion; LVSI, Lympho-vascular space invasion; EBRT, external beam radiation therapy; con-chemo, concurrent chemotherapy.

*p ≤0.05 in subgroup.

Compared with the superficial CSI group, more patients in the deep CSI group had deep myometrial invasion (32/44, 72.7% vs. 72/253, 28.5%, respectively; *P*<0.001), positive LVSI (19/44, 43.2% vs. 41/253, 16.2%; *P*<0.001) and received radiotherapy after surgery (35/44, 79.5% vs. 109/253, 43.1%, respectively; *P*<0.001). No differences were observed in the type of surgery, surgical approach, and tumor grade between the two groups.

### 3.2 Impact of varying depths of CSI on the prognosis of FIGO stage II EEC

**Figure 2:**
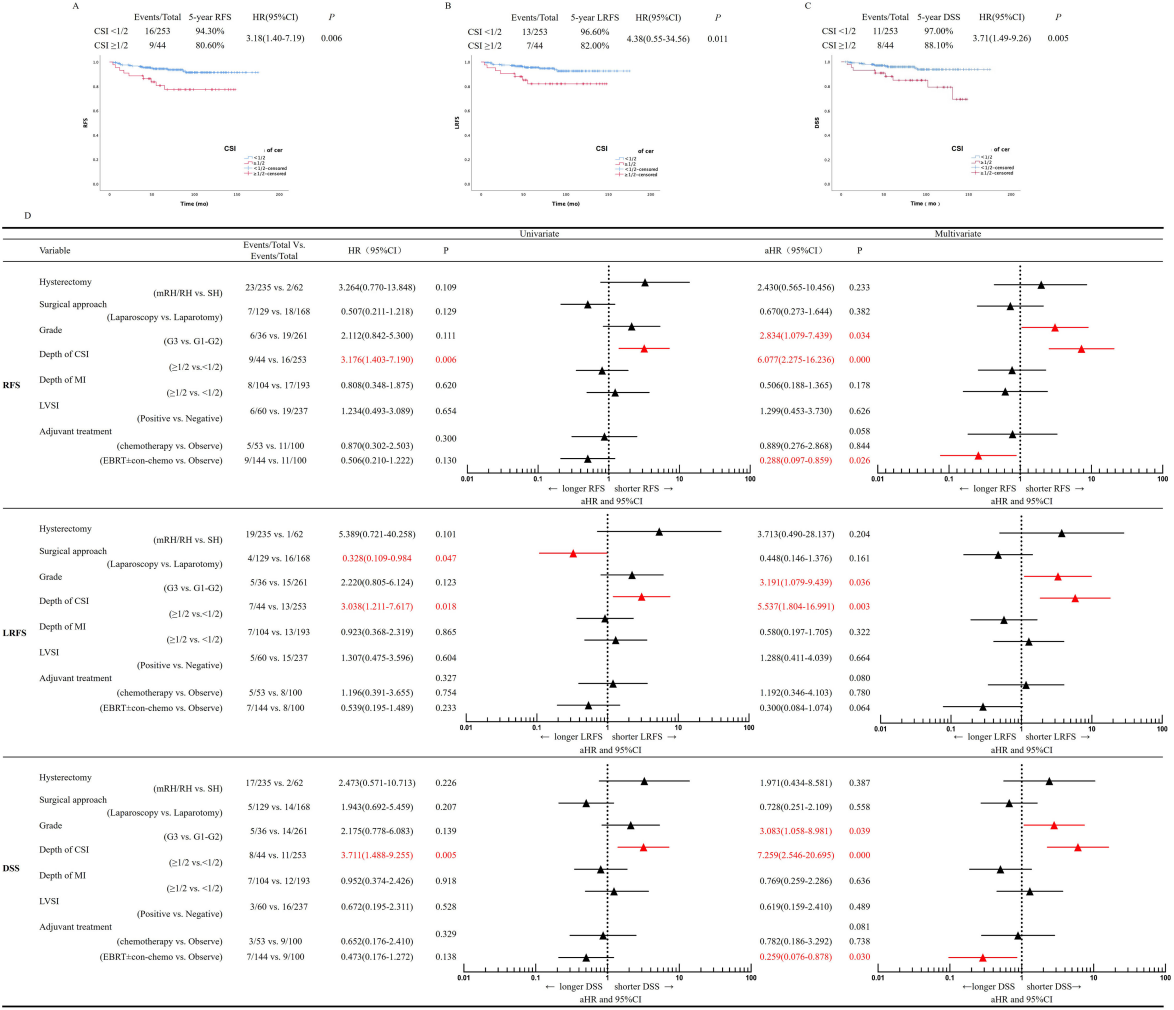
Univariate and multivariate regression analysis. **(A)** RFS, **(B)** LRFS, **(C)** DSS, according to depth of cervical stromal invasion modality in patients with International Federation of Gynecology and Obstetrics (FIGO) 2009 stage II EEC. **(D)** Multivariate analysis for RFS, LRFS and DSS. aHR, adjusted HR ratio, CI, confidence interval, DSS, disease–specific survival, EEC, endometrioid endometrial cancer, LRFS, locoregional recurrence–free survival, RFS, recurrence–free survival, CSI, cervical stromal invasion, MI, myometrial invasion, LVSI, Lympho–vascular space invasion, EBRT, external beam radiation therapy, co–chemo, concurrent chemotherapy.

There were 25 (25/297, 8.40%) recurrences. Patients were more likely to develop local pelvic recurrence than distant recurrence (20/297,6.73% vs. 5/297,1.67%, *P*=0.000). In the 20 locoregional recurrent cases, only one recurred at the vaginal vault, and the other 19 recurred in the pelvic cavity (8 cases were retroperitoneal lymph node recurrence, and the other 11 cases were pelvic masses). The 5-year RFS is 94.30% in the <50% CSI and 80.60% in the ≥50% CSI group, with a significant statistical difference between groups (*P*=0.006) (**Figure 2A**). The 5-year LRFS was 96.60% in the <50% CSI and 82.00% in ≥50% CSI group, with a statistically significant difference (*P*=0.011) (**Figure 2B**).

A total of 22 deaths (22/297, 7.4%) occurred, of which 19 (19/297, 6.4%) were uterine cancer related, three were died of other causes (one died of diabetes, one of cerebral infarction, and one of heart attack). Twelve patients (12/253, 4.7%) in the <50% CSI and 10 (10/44, 22.7%) in the ≥50% CSI group died from EC, with a statistically significant difference between groups (*P*=0.000) (**Table 2**). The 5-year disease-specific survival (DSS) was 97.00% in the <50% CSI and 88.10% in the ≥50% CSI group, with a statistically significant difference between groups (*P*=0.005) (**Figure 2C**).

**Table 2:** Pattern of death and recurrence according to depth of cervical stroma invasion.

	Total (%) (N=297)	CSI < 1/2 (%) (N=253)	CSI ≥ 1/2 (%) (N=44)	*P*
Total recurrence	25 (8.40)	16 (6.32)	9 (20.45)	0.006
Local recurrence	20 (6.73)	**13 (5.14)**	**7 (15.91)**	**0.011**
Vaginal	1(0.3)	0(0.00)	1(2.30)	
Pelvic	19(6.40)	13(5.10)	6(13.60)	
Distant recurrence	5 (1.67)	3 (1.18)	2 (4.54)	0.199
Deaths	22(7.40)	12(4.70)	10(22.70)	0.000
Non-disease related	3(1.00)	**1(0.40)**	**2(4.50)**	**0.044**
Disease related	19(6.40)	**11(4.30)**	**8(18.20)**	**0.001**

Bold values, there is statistically significant differences between groups.

CSI, cervical stromal invasion.

### 3.3 Risk factors related to prognosis of FIGO stage II EEC

We analyzed the possible risk factors related to recurrence and survival of the patients. On univariate analysis, depth of CSI was correlated with worse RFS, while the type of hysterectomy, surgical approach (laparoscopic vs. laparotomy), tumor grade, depth of myometrial invasion, LVSI, and adjuvant treatment were not found to be related to RFS. On multivariate Cox regression analysis, deep CSI (*P*=0.000, adjusted hazard ratio [aHR]=6.077, 95% CI: 2.275-16.236) and Grade 3. (*P*=0.034, aHR=2.834, 95% CI: 1.079-7.439) were independent predictive factors for poor RFS prognosis. Radiotherapy, with or without chemotherapy (Platinum-based concurrent chemotherapy), was observed to be independently related to longer RFS (*P*=0.026, aHR=0.288, 95% CI: 0.097-0.859) (**Figure 2D**).

On univariate analysis, depth of CSI and the surgical approach were predictors of a worse prognosis for LRFS. On multivariate Cox regression analysis, deep CSI (*P*=0.003, aHR=5.537, 95% CI: 1.804-16.991) and Grade 3. (*P*=0.036, aHR=3.191, 95% CI: 1.079-9.439) were found to be independent risk factors of LRFS.

On univariate analysis, depth of CSI correlated with a poor DSS prognosis, while the type of hysterectomy, surgical approach, tumor grade, LVSI, and adjuvant treatment did not correlate with DSS. On multivariate analysis, Grade 3. (*P*=0.039, aHR=3.083, 95% CI: 1.058-8.981) and deep CSI (*P*<0.001, aHR=7.259, 95% CI: 2.546-20.695) were identified as independent risk factors for a poor DSS prognosis (**Figure 2D**). Compared with observation, radiotherapy with or without concurrent chemotherapy was found to be independently related to DSS (*P*=0.030, aHR=0.259, 95% CI=0.076-0.878). The surgical approach (laparoscopic vs. laparotomy) did not affect DSS (*P*=0.558, aHR=0.728, 95% CI: 0.251-2.109) (**Figure 2D**).

## 4 Discussion

### 4.1 Summary of main results

In this two-centered retrospective analysis, we found that patients with FIGO stage II EEC were more likely to experience a local regional recurrence. Patients with deep CSI had a worse prognosis (RFS, LRFS and DSS) compared to patients with FIGO stage II EEC superficial CSI. Deep CSI and Grade 3 were independent risk factors for LRFS, RFS and DSS. Postoperative adjuvant radiotherapy (EBRT) was correlated with an improved prognosis for FIGO stage II EEC, while more extensive surgery such as mRH/RH was not correlated with a better prognosis.

### 4.2 Results in the context of published literature

CSI has been considered as an indicator of poor prognosis for patients with EEC (13, 23). However, when we stratified the patients with respect to the depth of CSI, those who had deeper CSI had an even worse prognosis, with a lower OS, RFS and LRFS. Previous studies have also reported similar results. Ferriss et al. included 85 cases of FIGO stage II EEC and found deep (inner two-thirds vs. superficial or outer one-third) CSI to be an independent predictor of death (HR 2.8; CI 1.1–7.2) (11). However, others have observed that women with EC characterized by deeper CSI did not have different survival or recurrence outcomes (13, 15, 16). Sondos et al. found no difference in the risk of recurrence or death between groups with <50% or ≥50% CSI in 117 patients with FIGO stage II EEC (16). These differences between various studies may be due to variability in how the cutoff value of depth of CSI was defined and the selection bias of retrospective studies.

Our study showed that pelvic recurrence was characteristic of patients with FIGO stage II EEC. Among the 25 patients with recurrence, 76.00% (19/25) displayed pelvic recurrence. EBRT with or without VBT is commonly recommended for FIGO stage II EEC disease (8, 23–25). Our findings confirmed that postoperative EBRT can improve prognosis of patients with FIGO stage II EEC. It should be noted that only one patient (1/25, 4.00%) experienced vaginal recurrence and none of our patients received VBT. Our results indicated that the addition VBT might not be needed for FIGO stage II EEC.

Our findings are consistent with other reports and have added new evidence showing that larger surgical extension using mRH or RH does not improve prognosis in FIGO stage II EEC. In our study, the 5–year OS in the mRH/RH group was 83%, compared with 80% in the SH group, and this difference was not statistically significant. Our data are consistent with other reports showing no prognostic benefit in patients receiving RH in FIGO 2009 stage II EEC (6, 26, 27). A systematic review in 2019 found no benefit of RH for the OS or disease–free survival in 2,866 patients with FIGO stage II disease (28). Therefore, SH should be the standard type of hysterectomy in FIGO stage II EEC to reduce surgical injury without compromising prognosis.

Based on two randomized trials (LACE (29) and LAP2 (30)), minimal invasive surgery has evolved into the surgical standard in early staged EC including “high–risk” patients. Daniel et al. (31) reported that a minimally invasive surgical approach in FIGO stage II EC is associated with higher recurrence rates and impaired clinical outcomes (HR 8.86 (1.008–20.85) and HR 6.36 (1.102–28.61), respectively). These data can be interpreted to be in line with the results of the LACC trial in cervical cancer. In our study, the surgical approach (laparoscopy vs. laparotomy) did not affect recurrence rates or clinical outcomes (**Figure 2D**), and this supports the use of a minimally invasive surgical approach in FIGO stage II EEC. Minimally invasive surgery allows for faster patient recovery, reduced bleeding, smaller incision sites, and better pain relief than laparotomy (32, 33). However, laparoscopic surgery requires advanced skills and experience from the surgeon, demands specialized equipment, limited visual field, et al. These limitations and challenges of laparoscopic surgery, which must be weighed against its advantages when making treatment decisions.

### 4.3 Strengths and weaknesses

Our study’s strengths include a relatively large cohort of consecutive stage II EEC patients. The median follow–up in our cohort was 75.0 months, which is in line or longer, than other published data (11, 16). Our study has some limitations. The addition of molecular classification to clinicopathological features can dramatically modify the risk class of a patient and consequently the adjuvant treatment (34–37). The patients included in this study were those between January 2008 and December 2017, who did not undergo molecular classification, neither POLE gene hotspot mutation–testing. Therefore, the relationship between molecular subtypes and prognosis was not analyzed. However, in our subsequent study, we included patients who had undergone comprehensive molecular profiling, allowing us to analyze the impact on prognosis.

As a retrospective study, we acknowledge the possibility of selection bias, particularly if individuals with missing data or certain characteristics were more likely to be excluded.

One key limitation of this study is the absence of a formal sample size calculation and the unequal number of cases between the two groups. As a retrospective observational study, the conclusions in the published literature regarding whether the depth of cervical stromal invasion in endometrial cancer affects prognosis are inconsistent. Therefore, the effect size (Δ) cannot be determined, making it hard to calculate the sample size satisfactorily. Future studies should prioritize calculating the sample size based on expected effect sizes and desired statistical power.

### 4.4 Implications for practice and future research

Our study has provided new evidence supporting the clinical implications of depth of CSI in patients with FIGO stage II EEC. A detailed pathological report about the depth of CSI is suggested to help better understand the prognosis and choose an appropriate postoperative treatment for the patient. Multi–centered prospective and larger studies are warranted to confirm the impact of depth of CSI on patients with FIGO stage II EEC.

## 5 Conclusions

In conclusion, this study showed that deep CSI has a poorer prognosis compared to superficial CSI in patients with FIGO stage II EEC. The recurrence location in our patient cohort was predominantly in the pelvic region. Postoperative EBRT can improve OS and RFS of these patients while VBT may not be needed. A detailed pathological report about the depth of CSI is suggested to help better understand the prognosis and choose an appropriate postoperative treatment for the patient.

## Data Availability

The raw data supporting the conclusions of this article will be made available by the authors, without undue reservation.
